# ssys: Exact algebraic recasting of ODE models into S-system or GMA form

**Published:** 2026-07-06

**Authors:** William S. Hlavacek

## Summary

ssys is a Python toolkit for exact algebraic transformation of ordinary differential equation (ODE) models into S-system or Generalized Mass Action (GMA) form. Given a model in Antimony format (Heydarabadipour et al., 2026; Smith et al., 2009), or an SBML file (Keating et al., 2020) parsed with ssys.parse_sbml, ssys produces a mathematically equivalent representation where each equation has one of the following forms:

**S-system form** (difference of two monomials):

(1) $${\dot{X}}_{i}=\alpha_{i}\prod_{j}X_{j}^{g_{ij}}-\beta_{i}\prod_{j}X_{j}^{h_{ij}},\;\alpha_{i},\beta_{i}\geq 0.$$

Equation 1 is a relaxed S-system form that permits zero-valued right-hand-side terms; canonical S-system form requires both constant coefficients in every equation to be strictly positive.

**GMA form** (sum of monomials):

(2) $$\frac{dX_{i}}{dt}=\sum_{k}\gamma_{ik}\prod_{j}X_{j}^{f_{ijk}}.$$

The transformation introduces auxiliary variables as needed to decompose a broad class of nonlinearities—including rational functions, exponentials, logarithms, sine, and cosine—into products of power-law terms. The recast is exact: the original and transformed systems have identical dynamics on the invariant constraint manifold defined by auxiliary variable definitions, given consistent initial conditions. Exactness is local to trajectories in the admissible real domain: denominators must avoid zero, logarithm arguments must remain positive, square-root arguments must remain valid, lifted variables used as power-law bases must remain positive, and auxiliary initial conditions must satisfy their defining relations.

The software provides a command-line interface (CLI) for batch processing Antimony models, a three-test validation suite (symbolic, numerical, trajectory), and generates Jupyter notebooks (Kluyver et al., 2016) with LaTeX renderings and trajectory comparisons. It converts Antimony models to SBML with the Antimony library (Heydarabadipour et al., 2026; Smith et al., 2009), parses the resulting SBML with libSBML (Bornstein et al., 2008), and exposes direct SBML-file parsing through ssys.parse_sbml.

### Statement of need

S-systems and GMA systems are canonical ODE forms developed within Biochemical Systems Theory (BST) (Savageau, 1976; Savageau & Voit, 1987). Savageau & Voit (1987) proved that systems built from sums, products, and compositions of elementary functions can be recast into S-system form; their theorem provides a minimum estimate of the class of recastable systems. These canonical representations offer several advantages: S-system steady-state equations become linear in log-space, enabling algebraic analysis of identifiability (Villaverde, 2019) and sensitivity (Savageau, 1971); the uniform power-law structure facilitates parameter estimation via linear regression techniques (Daniels & Nemenman, 2015b); and exact recast models can serve as ground truth for validating structure-learning algorithms that infer governing equations from data (Daniels & Nemenman, 2015a).

Despite the theoretical utility of S-systems and GMA systems, no general-purpose, open-source tool previously existed to perform exact recasting of supported symbolic ODE models. ssys fills this gap by providing:

- **Exact transformation.** The recast is algebraically equivalent to the original on the constraint manifold, verified by symbolic differentiation and numerical comparison.
- **Broad applicability.** The tool handles rational functions, selected elementary functions including exp, log, sin, and cos, and explicit time dependence through systematic lifting. sin and cos are represented with positive offset auxiliaries; logarithmic lifts require positive arguments, and all lifted variables used as power-law bases must remain positive. ssys does not automatically translate variables that can be zero or negative, so users must preprocess those models into the positive orthant before recasting.
- **Standard formats.** Input/output in Antimony format ensures interoperability with SBML-based workflows and model repositories such as BioModels (Malik-Sheriff et al., 2020).
- **Validation infrastructure.** A three-test suite (symbolic Jacobian verification, pointwise numerical sampling, trajectory comparison) provides certificates of correctness.

The package enables researchers to convert published models into canonical form for analysis, create ground-truth benchmarks for structure-learning algorithms, and leverage the log-linear properties of S-systems for algebraic steady-state analyses.

### State of the field

Antimony is a human-readable modeling language for systems biology, with transparent interoperability with SBML. The S-system and GMA formalisms are classical (Savageau, 1976; Savageau & Voit, 1987; Voit & Kemp, 2025), but existing software support has focused on analysis, construction, or approximation rather than exact general-purpose recasting. Classical BST computational-analysis workflows focus on systems already expressed in S-system or GMA form (Voit, 2000). PLMaddon (Vera et al., 2007) can generate power-law approximations via Taylor expansion, but such local approximations differ fundamentally from exact recasting with auxiliary variables. More recently, BSTModelKit.jl (Vadhin & Varner, 2026) provides Julia tools for constructing and analyzing BST models, though it emphasizes model construction and analysis from declarative specifications rather than recasting supported symbolic ODE models from SBML/Antimony inputs. To the best of our knowledge, no existing open-source tool performs exact algebraic recasting from supported SBML/Antimony ODE models to S-system or GMA form with explicit constraint-manifold handling and validation certificates.

### Software design

ssys is organized as a parser, symbolic transformation engine, formatter, and validation layer. Antimony models are converted to SBML with the Antimony library and then parsed with libSBML; SBML files can be parsed directly with ssys.parse_sbml. Both paths produce SymPy (Meurer et al., 2017) symbolic expressions, which serve as the internal representation for exact algebraic rewriting.

The recaster applies local exact transformations: composite functions (exp, log, sin, cos, etc.) and rational denominators are lifted to auxiliary variables with chain-rule-derived ODEs, and sums of monomials are factored into products via pool-auxiliary construction. The simplified output mode allows for relaxed S-system form, whereas the canonical mode rewrites equations to a strict two-term S-system form. Inputs with unsupported SBML semantics, unsafe generated derivatives, or violated domain assumptions are rejected, reported as unsupported, or fail validation rather than being treated as validated recasts. Correctness is checked by three independent validators: symbolic Jacobian chain-rule verification, pointwise numerical sampling, and trajectory comparison via libRoadRunner simulation (Somogyi et al., 2015; Welsh et al., 2023).

### Quality control

The package includes 712 pytest-collected test cases covering parsing, recasting, validation, and CLI functionality. Of these, 11 are integration-marked test cases, including 8 slow manifest tests that entail transformation and validation of 117 handwritten Antimony models across two output modes. Most of these models were drawn from published examples used to introduce, solve, or analyze S-system and related power-law representations: the complete Savageau & Voit example set (Savageau & Voit, 1987), later S-system examples and applications (Irvine, 1988; Rust & Voit, 1990; Savageau, 1988, 1993; Voit, 1988a, 1988b, 1990, 1992, 1993), metabolic optimization and model-inference examples using power-law canonical forms (Daniels & Nemenman, 2015b; Marin-Sanguino et al., 2007; Pozo et al., 2011), and nonlinear dynamics or control examples that test polynomial and quasipolynomial transformations (Anghel et al., 2013; Anguelov et al., 2018; Hernández-Bermejo et al., 1998; Papachristodoulou & Prajna, 2005; Zhang et al., 2022).

### BioModels database benchmark

To assess applicability to real-world models, we applied ssys to ODE models from the BioModels database (Malik-Sheriff et al., 2020). The benchmark suite (included in the repository under biomodels_batch/) operates in three phases: (1) fetch SBML models from BioModels, filtering for ODE-based models; (2) identify transformation candidates by excluding models with unsupported features or more than 100 dynamic species, 200 reactions, or 500 parameters; and (3) batch transform the candidates. After filtering 1,644 SBML models downloaded from BioModels, 978 candidates remained. Of these, the transformation algorithm completed for 848 (86.7%), and numerical validation confirmed mathematical equivalence for 739 transformed models. Among the validated models, 366 achieved meaningful structural canonization: 282 models in general form were converted to GMA, 77 models in GMA form were converted to S-system form, and 7 models in general form were converted to S-system form. The remaining validated models were already in GMA or S-system form, or retained general form after exact rewriting. Unsuccessful cases were mostly due to unsupported model features, parsing or loading errors, complexity limits, or timeouts. Benchmark scripts and the tracked summary are in biomodels_batch/; rerunning the benchmark writes detailed outputs under biomodels_batch/results/.

### Research impact statement

ssys makes recasting construction (Savageau & Voit, 1987) available as a practical tool for SBML and Antimony models. The curated handwritten examples provide regression tests with known recasting targets, while the BioModels benchmark demonstrates that exact recasting can be applied at repository scale to hundreds of published ODE models.

### Availability

ssys is available on GitHub at https://github.com/lanl/ssys. Installation requires Python >= 3.10 (and < 3.13), SymPy, NumPy (Harris et al., 2020), libRoadRunner, and the Antimony library. The repository includes comprehensive documentation: theoretical background and recasting rules (RECASTING.md), test model descriptions (TEST_MODELS.md), and usage instructions (README.md).

Installation: pip install ssys. For a single-model command-line example, put the path to an Antimony file such as test_models1/m01_exp_decay.ant in a one-line manifest and run ssys-recast --manifest models.manifest --outdir out --validate.

### AI usage disclosure

Generative AI assistance was used during software review, debugging, benchmark analysis, bibliography checking, and manuscript editing. The author made the scientific, architectural, and editorial decisions, reviewed AI-assisted changes, ran verification tests and paper builds, and is responsible for the correctness and licensing of the submitted materials.
